# CRISPR/Cas Enzyme Catalysis in Liquid–Liquid Phase‐Separated Systems

**DOI:** 10.1002/advs.202407194

**Published:** 2024-11-22

**Authors:** Yaqin Zhang, Jianai Chen, Zhina Wu, Chenfei Zhao, Rui Wang, Zhiping Li, Jiasi Wang, Di Wang

**Affiliations:** ^1^ Department of Clinical Pharmacy The First Hospital of Jilin University Jilin University Changchun Jilin 130021 China; ^2^ School of Life Sciences Jilin University Changchun Jilin 130012 China; ^3^ Jilin Provincial Key Laboratory of Tooth Development and Bone Remodeling Department of Orthodontics Hospital of Stomatology Jilin University Changchun 130021 China; ^4^ Department of Physics and Astronomy University of Manchester Manchester M13 9PL UK; ^5^ Guangdong Provincial Key Laboratory of Sensor Technology and Biomedical Instrument School of Biomedical Engineering Shenzhen Campus of Sun Yat‐sen University Shenzhen Guangdong 518107 China

**Keywords:** ATPS, Cas12a, Cas13a, CRISPR

## Abstract

The clustered regularly interspaced palindromic repeats (CRISPR) /CRISPR‐associated proteins (Cas) system is the immune system in bacteria and archaea and has been extensively applied as a critical tool in bioengineering. Investigation of the mechanisms of catalysis of CRISPR/Cas systems in intracellular environments is essential for understanding the underlying catalytic mechanisms and advancing CRISPR‐based technologies. Here, the catalysis mechanisms of CRISPR/Cas systems are investigated in an aqueous two‐phase system (ATPS) comprising PEG and dextran, which simulated the intracellular environment. The findings revealed that nucleic acids and proteins tended to be distributed in the dextran‐rich phase. The results demonstrated that the cis‐cleavage activity of Cas12a is enhanced in the ATPS, while its trans‐cleavage activity is suppressed, and this finding is further validated using Cas13a. Further analysis by increasing the concentration of the DNA reporter revealed that this phenomenon is not attributed to the slow diffusion of the reporter, and explained why Cas12a and Cas13a do not randomly cleave nucleic acids in the intracellular compartment. The study provides novel insights into the catalytic mechanisms of CRISPR/Cas systems under physiological conditions and may contribute to the development of CRISPR‐based molecular biological tools.

## Graphical abstract

The catalysis mechanisms of CRISPR/Cas systems are investigated in the LLPS which simulated the intracellular environment. It is found that the cis‐cleavage activity of Cas12a and Cas13a is enhanced in LLPS, while the trans‐cleavage activity is suppressed, which may contribute to the development of CRISPR‐based molecular tools.

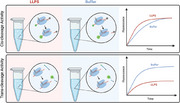

## 1 Introduction

The clustered regularly interspaced palindromic repeats (CRISPR) /CRISPR‐associated proteins (Cas) system functions as an adaptive immune system in bacteria and archaea by recognizing nucleic acids and degrading foreign nucleic acids.^[^ ^1^ ^]^ Cas9, Cas12a, and Cas13a proteins are effector nucleases belonging to type II, V, and VI CRISPR systems, respectively. These proteins facilitate the crRNA‐mediated cleavage of target nucleic acids by recognizing the complementary base pairs between the target nucleic acid and the crRNA. The crRNA then hybridizes with the target sequence to form a double strand, while leaving the non‐target strand (NTS) in an unbound state. The binding of the crRNA with the target strand (TS) activates the cis‐cleavage activity of Cas, which subsequently catalyzes the cleavage of the target nucleic acid.^[^ ^2^ ^]^

Despite functional similarities, Cas9, Cas12a, and Cas13a have distinct mechanistic characteristics. For instance, the Cas13a requires a protospacer flanking sequence (PFS), similar to the protospacer adjacent motif (PAM) sequence, which comprises a single A, U, or C base.^[^ ^3^ ^]^ Additionally, Cas9 and Cas12a are known to target DNA, while Cas13a targets RNA. In addition to the inherent cis‐cleavage activity of Cas proteins, Cas12a and Cas13a possess trans‐cleavage activity that enables them to cleave single‐stranded non‐target nucleic acids following activation.

The discovery of the trans‐cleavage activity indicates that Cas12a and Cas13a can be used for developing potential next‐generation molecular diagnostic techniques.^[^ ^4^ ^]^ CRISPR/Cas systems have been extensively applied in conjunction with various signal detection systems, including fluorescence,^[^ ^5^ ^]^ colorimetric,^[^ ^6^ ^]^ and electrochemical,^[^ ^7^ ^]^ CRISPR/Cas systems have been widely applied to construct novel biosensors for detecting nucleic acid and non‐nucleic acid targets. CRISPR/Cas systems have also been explored for applications in gene editing in vivo. Nevertheless, it is necessary to study the catalytic processes of CRISPR/Cas systems in an intracellular environment for understanding the mechanism underlying nucleic acid cleavage, and for enabling the further development of novel CRISPR‐based technologies. However, the in vivo catalytic behaviors of Cas12a and Cas13a proteins have rarely been investigated to date.

Cellular compartmentalization enables the separation of biomolecules, which facilitates the occurrence of thousands of biochemical reactions simultaneously.^[^ ^8^ ^]^ Intracellular compartmentalization is widely regarded as a critical organizing principle of life.^[^ ^9^ ^]^ A growing body of research suggests that non‐membrane organelles can support the compartmentalization of biological reactions similar to membrane‐bound compartments, which possibly reflects their transition from rich prebiotic chemistry to the earliest life forms.^[^ ^10^ ^]^ Non‐membranous organelles include subcellular bodies that lack a lipid boundary and have dimensions of 0.01–10 µm.^[^ ^11^ ^]^ Several intracellular non‐membrane organelles including nucleoli,^[^ ^12^ ^]^ stress granules,^[^ ^13^ ^]^ and P granules,^[^ ^14^ ^]^ can execute various biological functions.^[^ ^8^, ^15^ ^]^ Additionally, these membrane‐less organelles display the properties of liquids and are highly dynamic. These coacervates consist of proteins and nucleic acids, and can maintain their shape and dimensions for minutes or hours.^[^ ^16^ ^]^ They can also exchange substances with the surrounding environment for short periods of time, and regulate various biological activities, including RNA metabolism, ribosome biogenesis, and others, by facilitating the segregation of enzymes and other molecules.^[^ ^10b^ ^]^

Liquid‐liquid phase‐separated (LLPS) systems that support the formation of coacervates have been regarded as important model systems for studying non‐membranous organelles.^[^ ^17^ ^]^ Various oligomers and polymers can spontaneously form LLPS containing a high concentration of nucleic acids and other molecules in the liquid phase.^[^ ^18^ ^]^ These independent coacervates can facilitate biological reactions while interference from other molecules is due to the selective localization of enzymes and substrates within the coacervates.^[^ ^19^ ^]^ By studying an aqueous two‐phase system (ATPS) of polyethylene glycol (PEG) and dextran, Christopher A. Strulson et al. observed that the concentration of RNA in the dextran‐rich phase was 3000‐fold higher than that in the overall solution and that the rate of cleavage by ribozyme increased by 70 fold.^[^ ^20^ ^]^ The enzymes exhibit different catalysis behavior in LLPS.^[^ ^21^ ^]^

**Figure 1:**
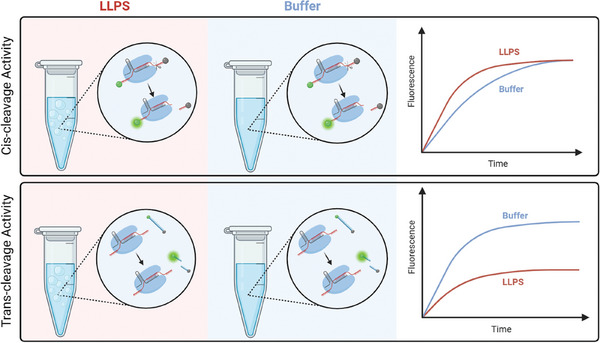
Schematic illustration showing the CRISPR/Cas12a catalysis system (cis‐cleavage and trans‐cleavage activity) in the ATPS.

The CRISPR‐Cas worked in the nucleus with the crowding of macromolecules.^[^ ^22^ ^]^ The trans and cis cleavage activities of Cas12a and Cas13a under this scenario remain poorly understood. In this report, we investigated the enzyme catalysis of the CRISPR/Cas12a and CRISPR/Cas13a systems in the ATPS system, a simple and widely used model to simulate the intracellular environment, formed by PEG/dextran. This system has been demonstrated that support simple biochemical reactions. We demonstrate that the cis‐cleavage activity of Cas12a was promoted and the trans‐cleavage activity of Cas12a was inhibited in the ATPS system (**Figure** **1**). Together, this work provides a model for the cis‐ and the trans‐acting DNase activities of CRISPR/Cas12a and CRISPR/Cas13a in intracellular conditions, and may contribute to developing novel CRISPR‐based biological tools.

## 2 Results and Discussion

### 2.1 Phase Separation of PEG and Dextran

**Figure 2:**
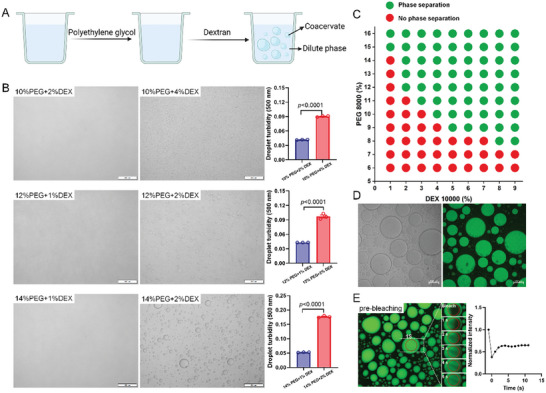
Formation of PEG and dextran coacervates. A) Schematic of coacervates approach. B) Brightfield images of the microdroplets prepared by dextran 10000 and PEG 8000. n = 3. C) Phase diagram of the PEG‐dextran phase‐separated droplets. D) Confocal microscopy images of droplets with PEG and FITC‐labeled dextran (10% PEG + 4% dextran). E) FRAP of dextran phase droplet in coacervate (10% PEG + 4% dextran).

The ATPS formed by the common neutral polymers, PEG (8 kDa) and dextran (10 kDa), was initially investigated in this study (**Figure** **2**A). The findings revealed that although both dextran and PEG are highly water soluble, the system spontaneously separates into two phases, a dextran‐rich droplet phase and a PEG‐rich continuous phase (Figure 2B). The complete phase diagram of this dextran‐PEG system is depicted in Figure 2C. The dextran was labeled with FITC to confirm the formation of the two separate phases. The results demonstrated that the dextran phase remained widely dispersed in water prior to the addition of PEG. However, the FITC‐labeled dextran formed a droplet phase following the addition of PEG, as observed by the green fluorescence (Figure 2D). The dextran phase was subsequently analyzed by FRAP experiments to obtain insights into the properties of the ATPS (Figure 2E). The corresponding fluorescence recovery profiles of the bleached area were obtained from the normalized FRAP data (Figure 2E). The recovery time was determined by fitting the recovery profiles to a double exponential model, which revealed that the ATPS formed by PEG and dextran exhibited the properties of the liquid. This indicates that biomolecules can flow in ATPS, and biochemical reactions can occur. The diffusion coefficients and viscosity of different phases were estimated based on the time of recovery. The diffusion coefficient for dextran was 1600 µm^2^ s^−1^, and the viscosity of dextran was 1.04 µPa.s.

### 2.2 Enrichment of Nucleic Acids and Proteins in the Dextran‐Rich Phase

**Figure 3:**
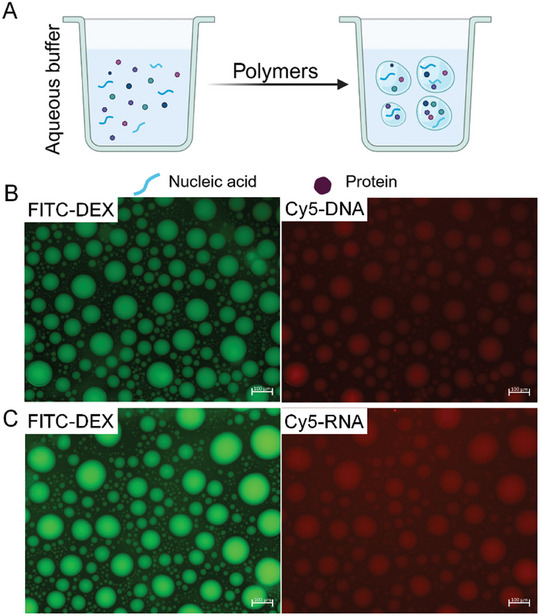
Localization of DNA and RNA in the two‐phase systems. A) Schematic depicting the distribution of DNA and RNA in the ATPS. B) Images of the dextran‐rich microdroplets (green fluorescence) and the Cy5‐labeled DNA (red fluorescence), were obtained by confocal fluorescence microscopy. C) Confocal fluorescence images of the dextran‐rich microdroplet (green fluorescence) and the Cy5‐labeled RNA (red fluorescence).

As living cells are abundant in proteins and nucleic acids, we analyzed the distribution of nucleic acids and proteins within the ATPS and determined the localization of DNA and RNA in the system (**Figure** **3**A). Visual characterization of the proteins and nucleic acids was achieved by labeling the DNA and RNA with Cy5 (650 nm), and the protein and dextran were labeled with rhodamine B isothiocyanate (RBITC) (595 nm) and FITC (520 nm) respectively. The results of fluorescence imaging revealed that the DNA, RNA, and protein tend to distribute in the dextran‐rich phase after mixing with PEG and dextran (Figure 3B,C; Figure S1, Suppporting Information).

### 2.3 Cis‐ and Trans‐Cleavage Activities of Cas12a in the ATPS

**Figure 4:**
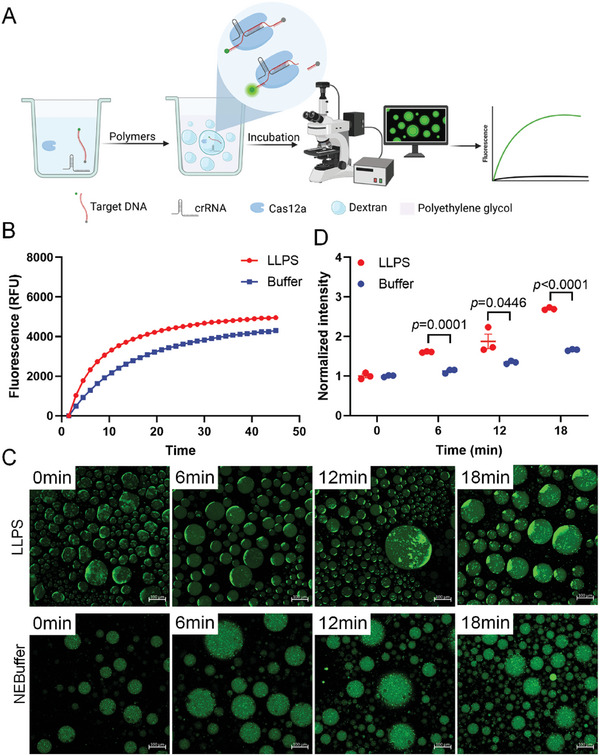
Detection of the cis‐cleavage activity of Cas12a in the ATPS. A) Workflow for detecting the cis‐cleavage activity of Cas12a. B) Comparison of the fluorescence growth rates in the buffer and ATPS. n = 3. C) Fluorescence micrographs for detecting the cis‐cleavage activity of Cas12a in the buffer and ATPS. D) Results of fluorescence quantification obtained in (C). n = 3.

The prokaryotic CRISPR‐Cas system functions as a programmable immune mechanism that employs crRNAs as guiding molecules for recognizing and targeting invasive nucleic acids, including viral DNA.^[^ ^23^ ^]^ The enzymes in the Cas12‐family possess two modes of DNase activity, namely, cis‐cleavage and trans‐cleavage activities.^[^ ^4^ ^]^ Briefly, Cas12a was pre‐combined with the crRNA in this study, following which the TS of the target dsDNA was allowed to hybridize with the crRNA. The complete R‐loop subsequently formed following the hybridization of crRNA‐TS and the unwinding of the TS‐ NTS. The structural domains of Cas12a were modified, and its cis‐ and trans‐cleavage activities were activated.^[^ ^24^ ^]^ We subsequently investigated the reactions catalyzed by CRISPR/Cas in the ATPS formed by PEG and dextran. As the DNA, RNA, and proteins were preferentially enriched in the dextran‐rich phase (Figure 3), it could be inferred that the enzymatic reactions catalyzed by CRISPR/Cas occur in the dextran‐rich compartments. We subsequently determined the activity of CRISPR/Cas12 by detecting the fluorescence signals in the dextran‐rich phase droplets (**Figure** **4**A). To this end, the cis‐cleavage activity of CRISPR/Cas12 was initially determined in this study. To confirm that the cis‐cleavage reaction catalyzed by CRISPR/Cas12 does occur within the phase‐separated dextran‐rich droplets, we designed a modified target DNA tagged with a fluorescent moiety and a quencher moiety at the 5′ and 3′ ends, respectively, for studying the localization and cleavage of target DNA. The cis‐cleavage reactions catalyzed by CRISPR/Cas12 were performed in both the buffer and the ATPS, and the fluorescent signals were measured using a real‐time PCR machine. It was observed that the fluorescence intensity in the buffer and ATPS increased over time, while the fluorescence intensity in the ATPS increased more significantly compared to that of the buffer (Figure 4B). The results demonstrated that the cis‐cleavage activity of Cas12 was activated in both the buffer and the ATPS (Figure 4B). The cis‐cleavage activity of CRISPR/Cas12 was subsequently measured using a confocal microscope to intuitively observe the fluorescence intensity in the droplets. The findings revealed that the final fluorescence intensity in the ATPS was higher than that in the buffer, indicating that the cis‐cleavage activity of Cas12a was enhanced in the ATPS (Figure 4C,D).

**Figure 5:**
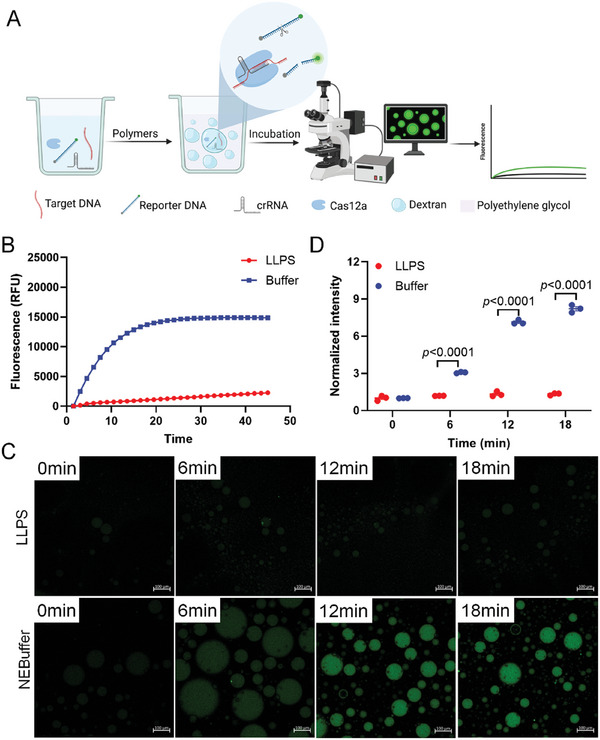
Detection of the trans‐cleavage activity of Cas12a in the ATPS. A) Workflow for detecting the trans‐cleavage activity of Cas12a. B) Comparison of the fluorescence growth rates in the buffer and ATPS. n = 3. C) Fluorescence micrographs for detecting the trans‐cleavage activity of Cas12a in the buffer and ATPS. D) The fluorescence quantification of (C). n = 3.

Based on these aforementioned observations, we further explored the trans‐cleavage activity of Cas12a in the ATPS (**Figure** **5**A). The results of real‐time fluorescence analyses revealed that the increase in fluorescence intensity was barely detectable in the ATPS group (Figure 5B). We then monitored the fluorescence in droplets with a confocal microscope. Compared with the buffer group, almost no fluorescence signal in the ATPS group could be observed (Figure 5C,D). The above phenomenon illustrates that the trans‐cleavage activity of Cas12a in ATPS was inhibited. It also shows that the fluorescence signals of Figure 4 in the ATPS group were almost all from the cis‐cleavage activity of Cas12a.

The findings revealed that the cis‐ and trans‐cleavage activities of Cas12a are dependent on the hybridization of the crRNA with the TS. We, therefore, speculated whether the hybridization of the crRNA with the TS affects the cis‐cleavage activity of Cas12a. In order to characterize the hybridization of the crRNA with the TS, a molecular beacon which was labeled by a fluorophore and a quencher on the ends of the DNA hairpin, was designed as the target DNA. The molecular beacon was designed in such a manner that the hairpin unfolds following hybridization with the crRNA, thereby enabling the measurement of the resulting fluorescence signals. The results of real‐time fluorescence analysis demonstrated that the fluorescence signals of the ATPS group increased more rapidly compared to that of the buffer group, and the fluorescence intensity was also higher in the ATPS group (Figure S2A, Suppporting Information). The results of fluorescence imaging additionally revealed that the molecular beacon in ATPS hybridized with crRNA more efficiently than that in the buffer group (Figure S2B,C, Suppporting Information). The results demonstrated that the binding rate of target DNA and crRNA in ATPS was faster than that in buffer, which might explain why the cis‐cleavage activity was enhanced in ATPS. Some studies indicate that molecular crowding can enhance the binding of nucleic acids,^[^ ^25^ ^]^ and our results align with this conclusion.

We hypothesized that the inhibition of the trans‐cleavage activity of Cas12a in the ATPS might be attributed to its higher viscosity, which reduced diffusion and consequently altered the contact efficiency between the reporter DNA and Cas12a. To test and validate this hypothesis, the concentration of the reporter DNA was increased to improve the contact efficiency. However, the trans‐cleavage activity of Cas12a remained undetectable even when the concentration of the reporter DNA was increased to 2 µM (Figure S3, Suppporting Information). This finding suggested that the trans‐cleavage activity of Cas12a is relatively low in vivo. The majority of the DNA in organisms is double‐stranded; however, a smaller portion can exist as quadruple helices and dynamic structures, which likely explains why Cas12a does not indiscriminately cleave other nucleic acids in vivo. The findings obtained herein demonstrate that it is possible that the trans‐cutting activity of Cas12a is inherently inhibited in intracellular environments so that the DNA intracellular is not randomly cut by Cas12a.

### 2.4 Cis‐ and Trans‐Cleavage Activities of Cas13a in the ATPS

To illustrate the universal applicability of our model system, the cis‐ and trans‐cleavage activities of another widely studied CRISPR/Cas protein, Cas13a were also investigated. The cis‐cleavage activity of Cas13a was initially investigated in this study. As expected, the intensity of the fluorescence signal in the ATPS than in the buffer (Figure S5, Suppporting Information), indicating that the cis‐cleavage activity of Cas13a was higher in the ATPS. The trans‐activity was then explored. Figure S6 (Suppporting Information) showed that there was almost no fluorescence signal observed in the ATPS group. This was consistent with the previous conclusion, indicating the low trans‐activity of Cas13a in cells.

## 3 Conclusion

The development of the CRISPR technology is currently at a critical juncture. Due to their specific nucleic acid recognition capabilities and precise base editing potential, CRISPR/Cas systems have emerged as a revolutionary toolkit for gene editing, and have been extensively applied in disease therapy and gene regulation. The discovery of novel CRISPR/Cas effectors has further enriched CRISPR systems, especially for Cas12 and Cas13. The discoveries of its trans‐cleavage activity made it a promising and powerful platform for next‐generation point‐of‐care testing (POCT) diagnostics. However, the trans‐cleavage activity intracellular appears to receive limited attention.

**Figure 6:**
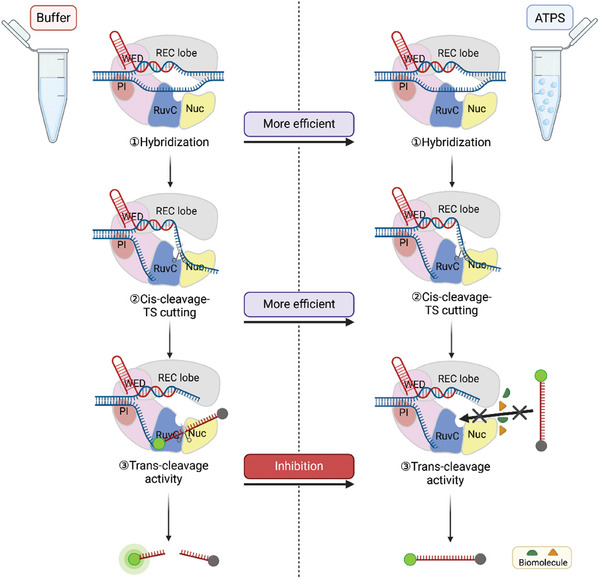
Schematic model of Cas12a‐mediated cis and trans cleavage of DNA in ATPS and buffer environment.

Coacervates formed by LLPS are an important model system for many membraneless organelles found in living cells.^[^ ^26^ ^]^ Studies have shown that the coacervates can undergo biochemical reactions and enhance the activity of some enzymes.^[^ ^20^, ^27^ ^]^ The present study investigated the catalytic behavior of Cas12 and Cas13 in the coacervate phase of ATPS formed by PEG/dextran, which was capable of simulating the intracellular environment (**Figure** **6**). The dextran‐rich phase was found to possess properties akin to liquids, and the proteins, RNA, and DNA tended to enrich in the dextran‐rich phase. Then, we explored the cis‐ and trans‐cleavage activity of Cas12a in the membrane‐less compartments. Cas12‐family enzymes demonstrate two distinct modes of DNase activity: in addition to crRNA‐guided cis‐cleavage of target DNA, these nucleases also facilitate trans‐cleavage of non‐target ssDNA substrates upon binding and allosteric activation by a target DNA. The Cas12a/crRNA complex identifies DNA targets via the PAM sequence, inducing the unwinding of the TS‐NTS duplex and facilitating the hybridization of crRNA‐TS. This hybridization triggers a conformational change in the REC, which exposes the RuvC catalytic site and promotes cis‐cleavage of both NTS and TS. We found that the cis‐cleavage activity of Cas12a was enhanced and the trans‐cleavage activity of Cas12a was inhibited in ATPS, which was further confirmed by using Cas13a.

The hybridization of crRNA‐TS DNA, rather than PAM binding perse, is the primary driver of allosteric activation of Cas12a by displacing the REC2 domain and thereby exposing the RuvC catalytic site.^[^ ^28^ ^]^ The enhanced cis‐cleavage activity could be attributed to the more efficient hybridization of crRNA with the TS in ATPS (Figure 6). Numerous studies have consistently demonstrated that the physical state of an enzyme's environment significantly influences its catalytic activity.^[^ ^29^ ^]^ As the interface pressure increases, the enzyme's activity tends to increase.^[^ ^30^ ^]^ This may also be a contributing factor to the enhancement of cis‐cleavage activity. The non‐target ssDNA reporter needs to interact with the catalytic site in the RuvC domain for the trans‐cleavage activity of Cas enzymes.^[^ ^28^ ^]^ We hypothesized that macromolecular overcrowding may prevent the reporter DNA from approaching the catalytic pocket, thus inhibiting the trans‐cleavage activity (Figure 6). We therefore attempted to reverse the inhibition of the trans‐cleavage activity by increasing the concentration of the reporter DNA. However, the trans‐cleavage activity of Cas12a remained undetectable even when the concentration of the reporter DNA was 2 µM. These findings indicated that the trans‐cleavage activity of Cas12a and Cas13a are inherently inhibited in intracellular environments. It helps to explain why the Cas12a and Cas13a will not damage the non‐target nucleic acids intracellularly. Our study may shed light to study the reaction mechanism of CRISPR/Cas in physiological conditions and may be useful for further development and utilization of Cas12a and Cas13a as molecular biological tools.

It should be admitted that the real environment within cells is complex, involving many proteins and nucleic acids. The ATPS formed by PEG and dextran cannot fully simulate the in vivo environment, and we can only draw preliminary conclusions in this work. Future studies with other models are needed to further explore the catalytic mechanism of CRISPR‐Cas in the intracellular environment.

## 4 Experimental Section

### Materials

All the DNA and RNA sequences, as well as the manufacturer information, are provided in Table S1 (Suppporting Information); PEG 8000, fluorescein isothiocyanate‐Dextran (FITC‐Dextran‐10 000) and Mineral oil were bought from Sigma–Aldrich (Shanghai, China); Dextran‐10 000 was obtained from Macklin (Shanghai, China); LbaCas12a and NEBuffer r2.1 were purchased from New England Biolabs (Shanghai, China); LwaCas13a was obtained from Edgene (Guangzhou, China). Bovine serum albumin (BSA, 66 kDa) was obtained from Sangon (Shanghai, China). Abil EM90 was purchased from Evonik (Shanghai, China); RBITC‐BSA (66 kDa) was purchased from Bioss (Beijing, China).

### Preparation and Characterization of the Coacervate

The coacervate samples consisted of PEG 8000 and Dextran 10 000 at different concentration ratios. The two polymers were dissolved in DEPC‐treated water. The turbidity of the sample mixtures was measured by monitoring the absorbance at 500 nm using a microplate reader (Epoch 2, Bio‐Tek, America).

(1) $$\mathrm{Turbidity}\;\left(\%\right)=100-10^{2-\left(\mathrm{Abs}\;500\;\mathrm{nm},\;\mathrm{sample}-\mathrm{Abs}\;500\;\mathrm{nm},\;\mathrm{buffer}\right)}$$

As turbidity alone cannot be used to distinguish aggregates from coacervate solutions, the presence of coacervate droplets was therefore subsequently investigated using an inverted optical microscope (AX10, ZEISS, Japan). The coacervate droplets were further characterized by adding 1% FITC‐Dextran to the mixture, which was subsequently shaken on a vortex mixer. Images of the samples were finally captured using a confocal microscope.

### Fluorescence Recovery after Photobleaching (FRAP)

The FITC‐labeled dextran droplets (10% PEG + 4% dextran, and all subsequent reactions were performed using this system) were further analyzed by FRAP experiments. The dextran phase was bleached using a 488 nm diode laser at 100% power. Imaging was performed using a 488 nm laser for excitation of the FITC dye (λ_FITC_ = 488 nm) with an emission wavelength (λ_FITC_ = 500–590 nm). Pre‐bleaching images were acquired for each experiment prior to bleaching, and fluorescence recovery was subsequently recorded by imaging in the FITC channel. According to the results of FRAP, the Diffusion coefficients (D) could be estimated:

(2) $$D=\frac{0.25{r_{\mathrm{BR}}}^{2}}{\tau_{1/2}}$$

where r_BR_ is the radius of the bleaching region and *τ*_1/2_ is the half‐time of recovery. Viscosity was calculated using the Stokes–Einstein equation:

(3) $$\eta =\frac{k_{B}T}{4\pi Dr_{\mathrm{DP}}}\;$$

where k_B_ is the Boltzmann's coefficient, T is the temperature, D is the diffusion coefficient and r_DP_ is the radius of the diffusing particle.

### Partitioning Experiments

Coacervate samples were prepared as described in Section 2.2 except that the same volume of Cy5‐labeled DNA was added instead of water to achieve a final concentration of 500 nM. For RNA partitioning experiments, Cy5‐labeled RNA (500 nM) was added to the coacervate. For protein partitioning experiments, 1% RBITC labeled BSA was added to the coacervate. Microscope images of the coacervate samples were captured using a laser confocal microscope (LSM710, ZEISS, Japan), and an inverted confocal microscope, at an excitation wavelength of 633 nm. Each of the experiments was repeated at least three times.

### Hybridization Potential of crRNA with a Molecular Beacon

The hybridization potential of the crRNA with the target DNA was monitored using a molecular beacon labeled with carboxyfluorescein (FAM) and black hole quencher 1 (BHQ1) labeled at the 5′ end and 3′ end, respectively. The molecular beacon was designed in such a manner that it unfolds in the presence of crRNA, which increases the distance between the ends of the molecular beacon, and the resulting fluorescence due to FAM can be subsequently monitored. However, the molecular beacon remains folded in the absence of crRNA, and no fluorescence signal can be detected. The final fluorescence was monitored by a real‐time PCR machine (fluorescence was recorded every 1.5 min) and a laser confocal microscope (the image was acquired every 3 min). For the buffer control group, the sample utilized the water‐in‐oil model (10% reaction mixture, 80% mineral oil, and 10% Abil EM90 (v/v)), and the fluorescence was observed in the aqueous phase.

### Cleavage Reactions Mediated by CRISPR/Cas12a and Cas13a

Coacervate samples were prepared as previously described in Section 2.2 except that the same volume of a CRISPR/Cas12a‐based cleavage reaction system was used instead of water. The cleavage reaction by CRISPR/Cas12a was performed according to the manufacturer's instructions. Briefly, the Cas12a and crRNA were pre‐incubated at 37 °C at the ratio of 1:1.25. The target DNA was modified by tagging a fluorescent group and a quenching group at the 3′ end and a quenching group at the 5′ end, respectively, for assessing the cis‐cleavage activity. The final cis‐cleavage reaction system consisted of 1 × NEBuffer r2.1 (50 mM NaCl, 10 mM Tris‐HCl, 10 mM MgCl_2_, and 100 µg mL^−1^ Recombinant Albumin, pH 7.9), 500 nM Cas12a, 625 nM crRNA, and 500 nM of the target DNA. For trans‐cleavage activity, the final reaction consisted of 1 × NEBuffer r2.1, 50 nM Cas12a, 62.5 nM crRNA, 10 nM of the target DNA, and 500 nM of the reporter DNA.

The cleavage activity of Cas13a was prepared according to the Cas12a, the reaction ratio was consistent, and the reaction buffer (50 mM NaCl, 40 mM Tris‐ HCl, 9 mM MgCl_2_, 1 mM DTT, pH 7.5) was different. The fluorescence acquisition was performed as above.

### Statistics

All data were presented as the mean ± S.E.M. The T‐test was then performed using SPSS 16.0 software (IBM Corporation, Armonk, NY). Statistical significance was considered when the *p*‐value was less than 0.05.

## Conflict of Interest

The authors declare no conflict of interest.

## Author Contributions

Y.Z. performed investigation, methodology, visualization, validation, formal analysis, and wrote the final manuscrpit. J.C. performed methodology, validation, investigation. Z.W. performed methodology, validation, investigation. C.Z. performed validation, investigation. J.W. performed conceptualization, visualization, supervision, wrote, reviewed and edited the final manuscrpit. Z.L. performed conceptualization, visualization, supervision. D.W. performed conceptualization, visualization, Funding acquisition, wrote, reviewed and edited the final manuscrpit. R.W. performed conceptualization, visualization, supervision.

## Supporting information



Supporting Information

## Data Availability

The data that support the findings of this study are available from the corresponding author upon reasonable request.
