# Supplementary material for: CRISPR/Cas Enzyme Catalysis in Liquid–Liquid Phase‐Separated Systems
Source: Adv Sci (Weinh). 2024 Nov 22;12(3):2407194. doi: 10.1002/advs.202407194 (PMC11744712; doi:10.1002/advs.202407194)
Supplement: Supplementary file 1 — Supporting Information [file ADVS-12-2407194-s001.docx]

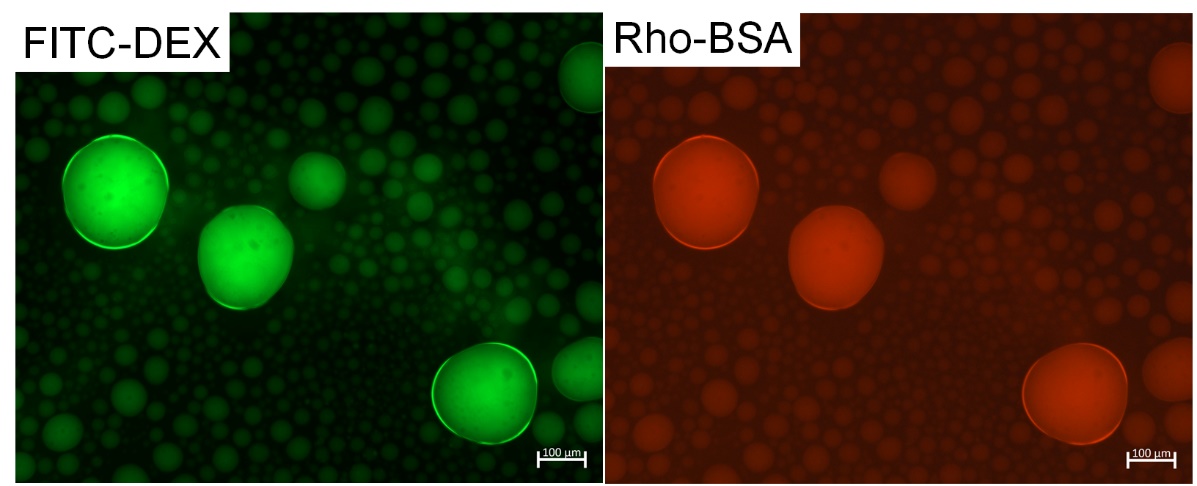


Figure S1. Confocal fluorescence images of the dextran-rich microdroplet (green fluorescence) and the Rho-labeled BSA (red fluorescence).


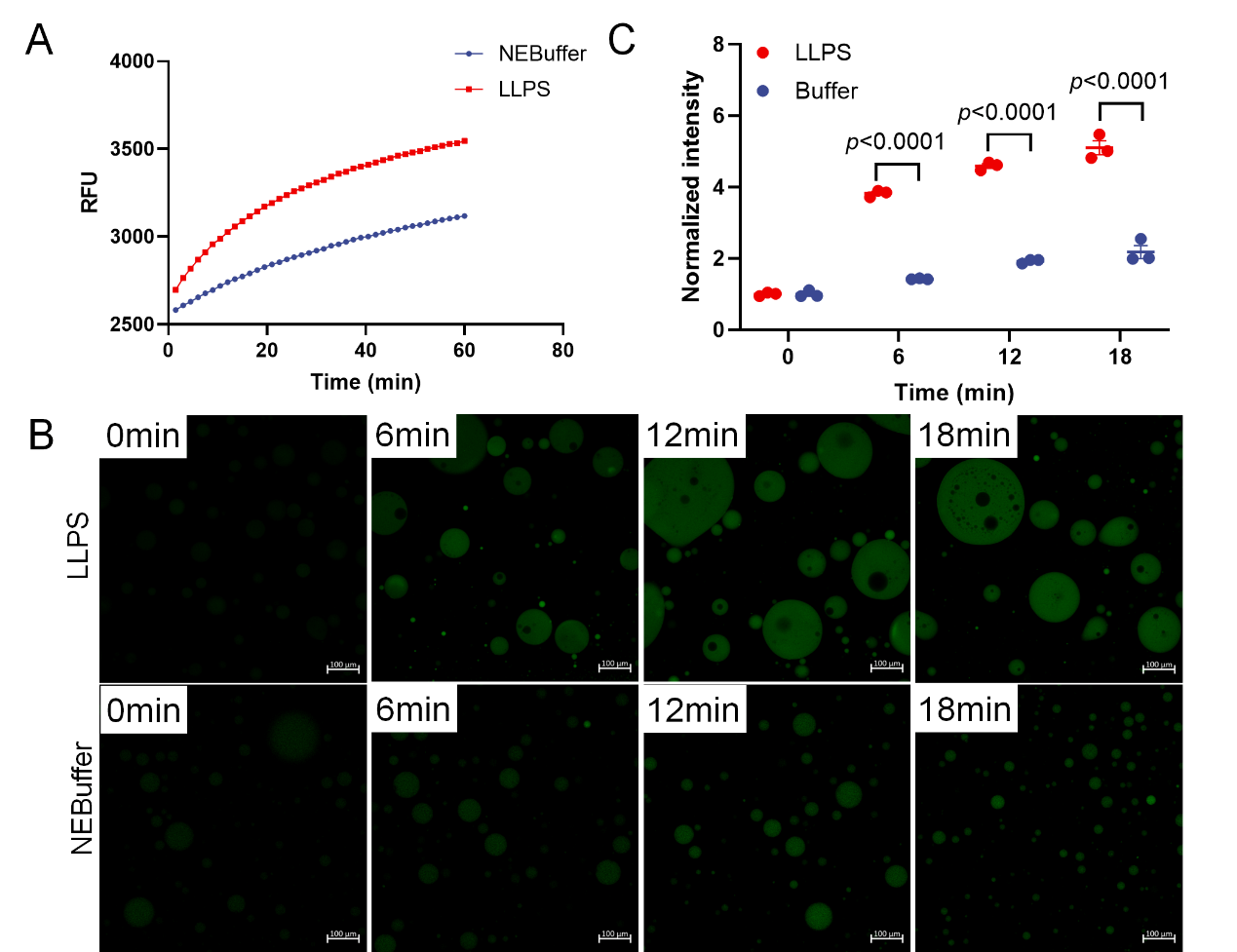


Figure S2. Detection of the hybridization of crRNA and molecular beacon in ATPS. (A) Real-time sensor fluorescence curve for detection in buffer and the ATPS. (B) Fluorescence micrographs for detection in buffer and the ATPS. (C) The fluorescence quantification of (B). n=3.


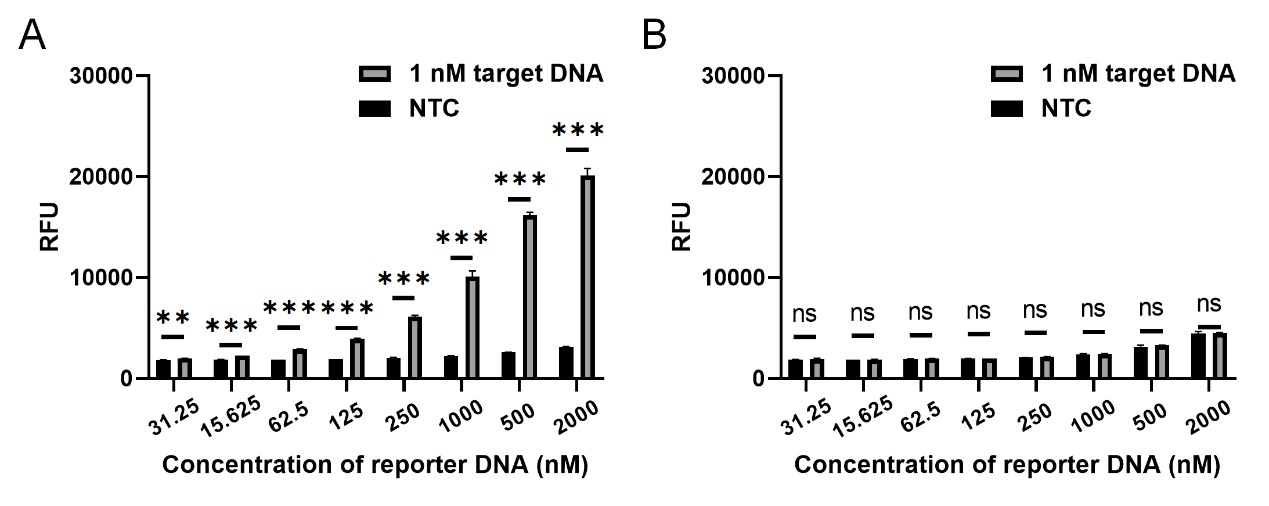


Figure S3. Sensor fluorescence for CRISPR/Cas12a trans-cleavage detection in buffer (A) and the ATPS (B) in different concentrations of reporter DNA. n=3.

Figure S4. The detection limit of CRISPR/Cas13 in the buffer. n=3.


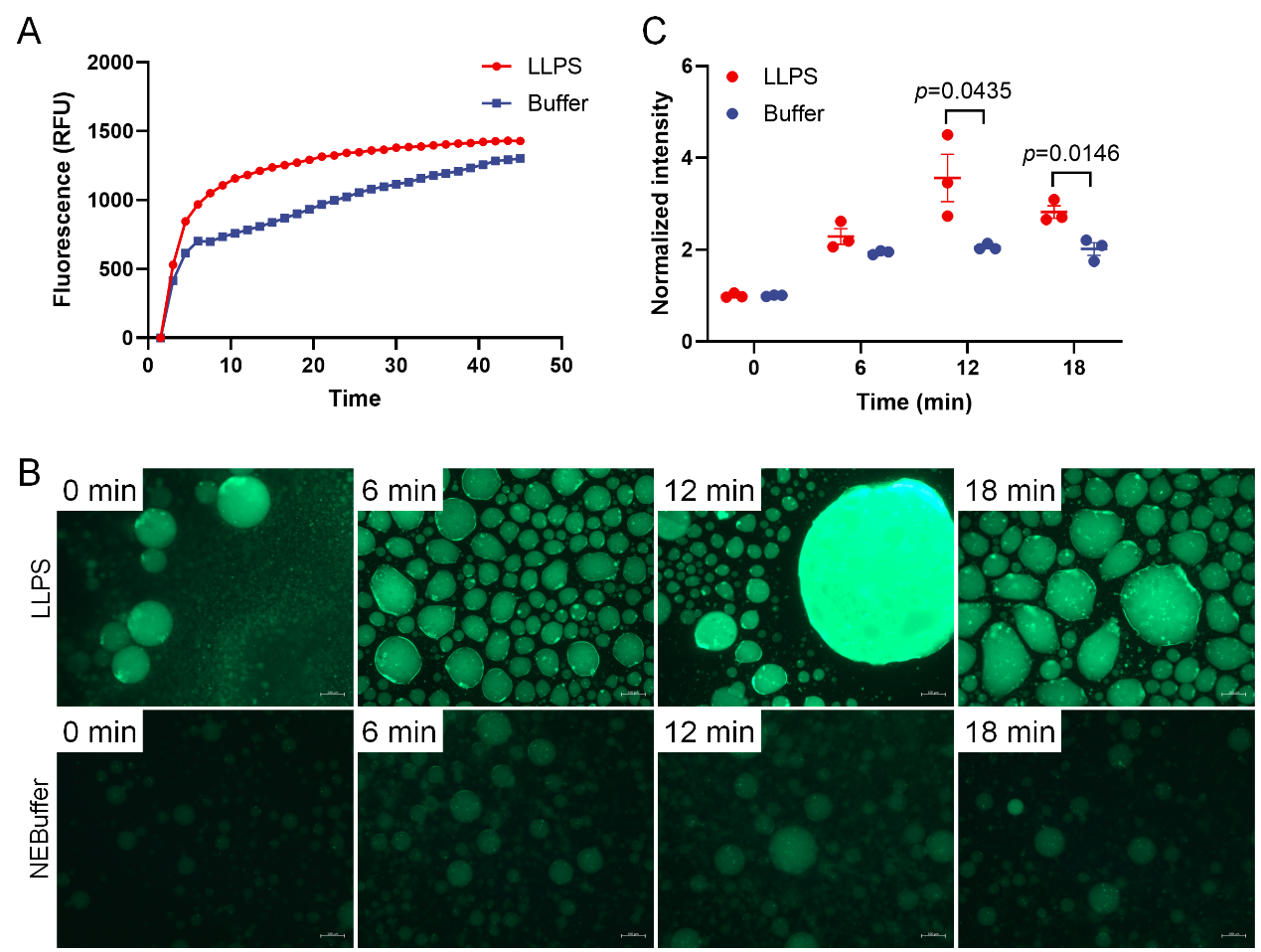


Figure S5. Detection of cis-cleavage activity of Cas13a in ATPS. (A) Comparison of fluorescence growth per unit of time in buffer and the ATPS. (B) Fluorescence micrographs for detection in buffer and the ATPS. (C) The fluorescence quantification of (B). n=3.


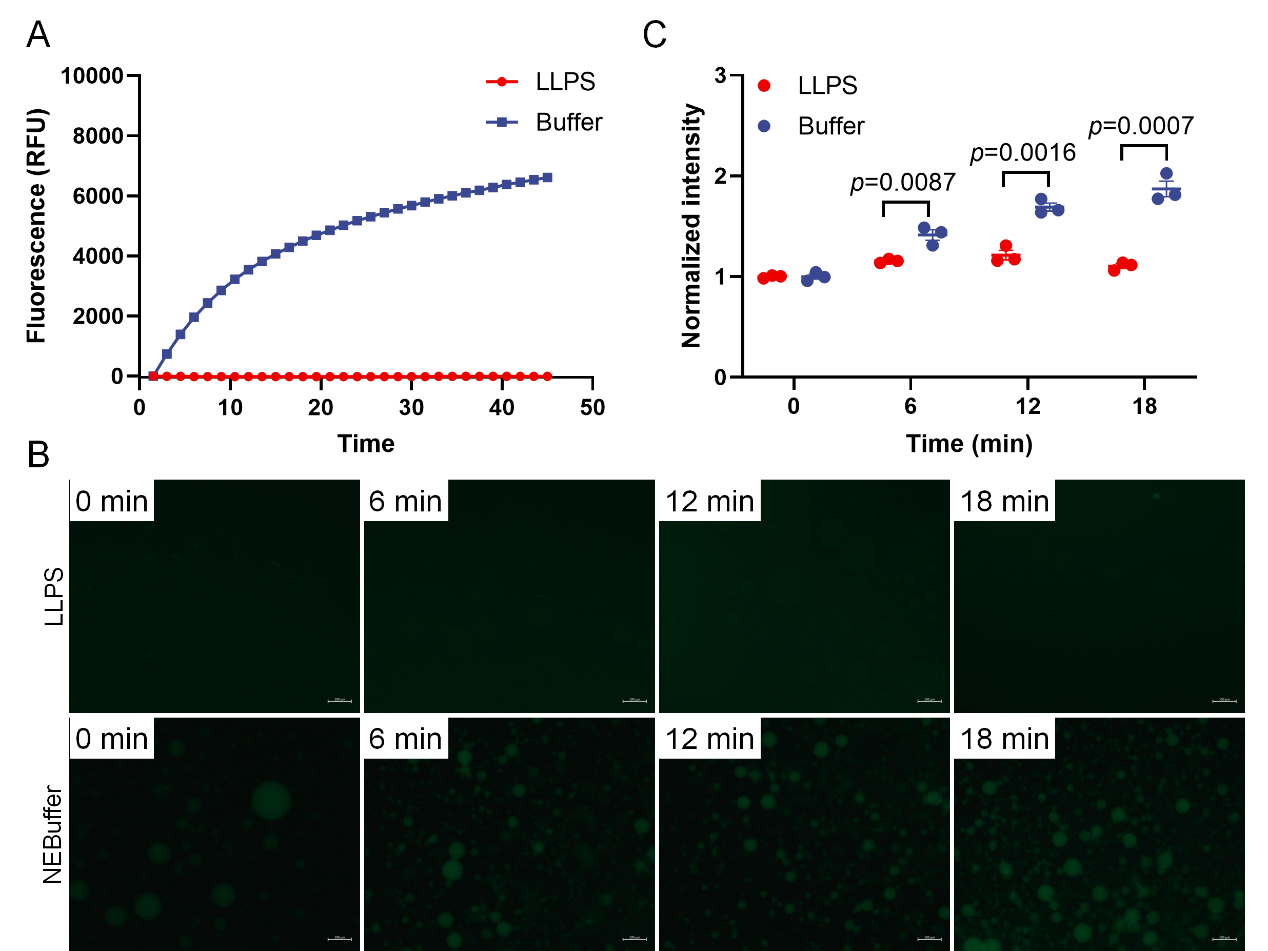


Figure S6. Detection of trans-cleavage activity of Cas13a in ATPS. (A) Comparison of fluorescence growth per unit of time in buffer and the ATPS. (B) Fluorescence micrographs for detection in buffer and the ATPS. (C) The fluorescence quantification of (B). n=3.

**Table S1** DNA and RNA sequences used in this study.

| Name | Sequence (from 5' to 3') |
| --- | --- |
| DNA-Cy5 | Cy5-TTTCTGTCATTC |
| RNA-Cy5 | Cy5-CUACCUUGUUCC |
| Target DNA | ATGCGCGACATTCCGAAGAACGCTGAAGCGCTGGGGGCAAATTGTGCAAT |
| crRNA-Cas12a | UAAUUUCUACUAAGUGUAGAUCCCCCAGCGCUUCAGCGUUC |
| cis-cleavage DNA | BHQ1-ACATTCCGAAGAACGCT-FAM-GAAGCGCTGGGGGCAAATTG |
| reporter-DNA | Cy5-TTTCTGTCATTC-BHQ3 |
| DNA hairpin | FAM-CCCCCTGAAGAACGCTGAAGCGCTGGGGG-Dabcyl |
| Target RNA | GCUAGAAUGGCUGGCAAUGGCGGUGAUGCUGCUCUUGCUUUGCUGCUGCUUGACAGA |
| crRNA-Cas13a | GAUUUAGACUACCCCAAAAACGAAGGGGACUAAAACAAAGCAAGAGCAGCAUCACCGCCAUUGC |
| cis-cleavage RNA | BHQ1-AGAAUGGCUGGCAAUGGCGGUGAUGCUGCUCUUGCUUUGC-FAM |
| reporter RNA | FAM-rUrUrUrUrUrU- BHQ1 |
